# Patient-specific breath-hold reproducibility in thoracic and abdominal radiotherapy: comparison of auditory and visual biofeedback

**DOI:** 10.1007/s11604-026-01948-0

**Published:** 2026-02-25

**Authors:** Masahide Saito, Naoki Sano, Ryota Tozuka, Hikaru Nemoto, Koji Ueda, Takafumi Komiyama, Kan Marino, Hiroshi Onishi

**Affiliations:** Department of Therapeutic Radiology, University of Yamanashi, 1110 Shimokato, Chuo, Yamanashi 409-3898 Japan

**Keywords:** Respiratory motion management, Audio-visual feedback, Breath-hold, Reproducibility

## Abstract

**Background and purpose:**

This study aimed to compare patient-specific reproducibility and comfort of breath-holding using auditory and visual biofeedback guidance in a respiratory monitoring system.

**Materials and methods:**

A respiratory monitoring device that tracks abdominal and chest wall motion was used to provide auditory and visual feedback. Forty patients with thoracic or abdominal tumors underwent three computed tomography scans under each guidance method. Breath-hold reproducibility was assessed by measuring the distance between an anatomical landmark and the tumor. Patient preferences for the guidance method were also recorded.

**Results:**

Both guidance methods demonstrated good breath-hold reproducibility. The mean displacement of the landmark-tumor distance was 1.26 ± 0.82 mm with visual guidance and 1.32 ± 0.72 mm with auditory guidance, with no statistically significant difference. No correlation was found between the two methods in individual patients. While 22 patients preferred visual guidance, 12 preferred auditory guidance, and 6 had no preference, the preferred method did not always correspond to improved reproducibility.

**Conclusion:**

Regarding the reproducibility of breath-holding, both auditory and visual guidance methods demonstrated high precision. However, no significant differences were observed between the two approaches, and patient preference did not appear to affect reproducibility.

## Introduction

In radiotherapy, precise management of each respiratory motion reduces the internal target volume and improves the therapeutic efficacy for abdominal and thoracic organs influenced by respiratory motion. Implementing this technique to reduce the internal target volume results in a smaller radiation field and decreased exposure to surrounding normal tissues. Consequently, this may lead to a reduction in treatment-related adverse events and potentially allow for dose intensification without compromising tumor control [1, 2]. Several respiratory control techniques have been developed, including breath-holding [3, 4], respiratory gating [5–7], and beam tracking [8, 9]. Although breath-holding requires individualized patient training and specialized equipment, it remains a widely adopted technique [10, 11]. Deep inspiration breath-hold (DIBH) has gained attention in recent years for its potential to reduce cardiac radiation dose during left breast cancer irradiation [12].

Breath-hold irradiation, while effective in mitigating the impact of respiratory motion during radiotherapy, places a considerable burden on clinical staff due to the additional time required for patient coaching, repeated verification of breath-hold reproducibility, and potential workflow disruption [13]. Notably, a subset of patients demonstrates difficulty maintaining consistent breath-hold levels under visual guidance alone, thereby compromising treatment precision. To overcome these limitations, our group previously developed a simplified respiratory monitoring system (Abches, APEX Medical Inc., Tokyo, Japan), which utilizes dual-point motion detection on the chest and abdominal walls [14]. This system allows patients to regulate their respiratory phase autonomously using either visual or auditory biofeedback during computed tomography (CT) simulation and treatment sessions.

In an earlier pilot study [15], we introduced an auditory-guided version of the system (Audio-Abches) and conducted a preliminary comparison between visual and auditory biofeedback modalities. However, that study was limited by a small sample size and possible confounding effects due to the fixed intervention sequence (e.g., auditory always followed by visual), which may have introduced learning or fatigue biases. Accordingly, the present study was undertaken to address these methodological limitations and to conduct a more comprehensive evaluation of breath-hold reproducibility and patient comfort under both visual and auditory biofeedback guidance using the updated Abches system.

## Methods and materials

### Study design

This study was approved by the institutional review board of the University of Yamanashi (approval number: 2969). Forty patients with thoracic or abdominal tumors participated. Each patient underwent three CT scans using both guidance methods. To assess breath-hold reproducibility, the distance between an anatomical landmark and the tumor position was measured. Additionally, patients were asked which guidance method they preferred for comfortable breath-holding. Patient characteristics are summarized in Table 1.

**Table 1:** Patient characteristics

Characteristic	Group A (Visual ⇒ Audio)	Group B (Audio ⇒ Visual)
N	20	20
Age [median (range)]	67 (54–85)	74 (65–88)
Sex [male/female]	13/7	17/3
Treatment site (Inhalation)	Lung: 10 (8 upper; 2 lower)	Lung: 18 (3 upper; 6 middle; 11 lower)
Treatment site (Exhalation)	Liver: 3 Pancreas: 3 Lymph node: 3 Kidney: 1	Lymph node: 2
Performance status [median (range)]	90 (80–100)	90 (80–100)

**Fig. 1:**
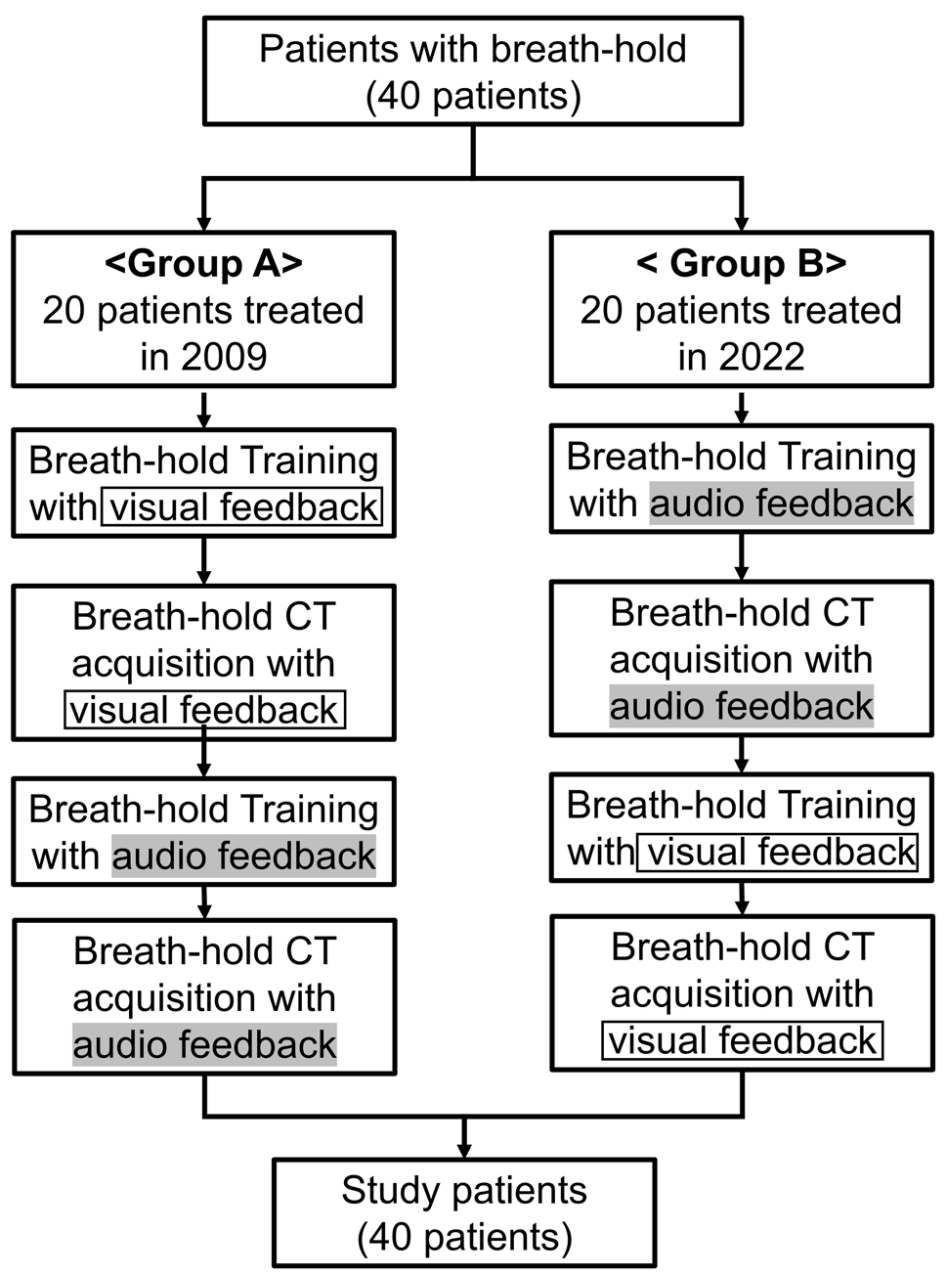
Flowchart of this study. Forty patients underwent breath-hold CT with both visual and audio feedback in different sequences to compare guidance

As illustrated in Fig. 1, this retrospective study included 40 patients who underwent breath-hold irradiation. The patients were divided into two cohorts according to the treatment period and the sequence of feedback modalities. Group A consisted of 20 consecutive patients treated in 2009, and Group B consisted of 20 consecutive patients treated in 2022. In Group A, patients first received breath-hold training using visual feedback, followed by CT image acquisition under the same visual feedback conditions. Subsequently, the same patients underwent breath-hold training with audio feedback, and a second CT scan was obtained using audio feedback guidance. In contrast, Group B followed the reverse order: patients initially received BH training with audio feedback and underwent CT acquisition under audio feedback conditions, after which breath-hold training and CT acquisition were repeated using visual feedback guidance. Thus, all 40 patients underwent CT simulations under both feedback modalities, enabling intra-patient comparison of visual versus audio feedback.

It should be noted that the two groups differed in both the study period and technical environment. Group A patients were treated in 2009 using an early-generation breath-hold monitoring system, whereas Group B patients were treated in 2022 with an upgraded system and refined protocols. Therefore, potential differences in imaging resolution, system performance, and operator experience were anticipated. To account for these temporal and technical variations, comparative analyses were performed within each group (visual vs. audio feedback) and between groups (Group A vs. Group B) to evaluate the reproducibility and accuracy of breath-hold guidance and to assess the impact of system evolution over the 13-year interval.

### Details of auditory and visual feedback system

**Fig. 2:**
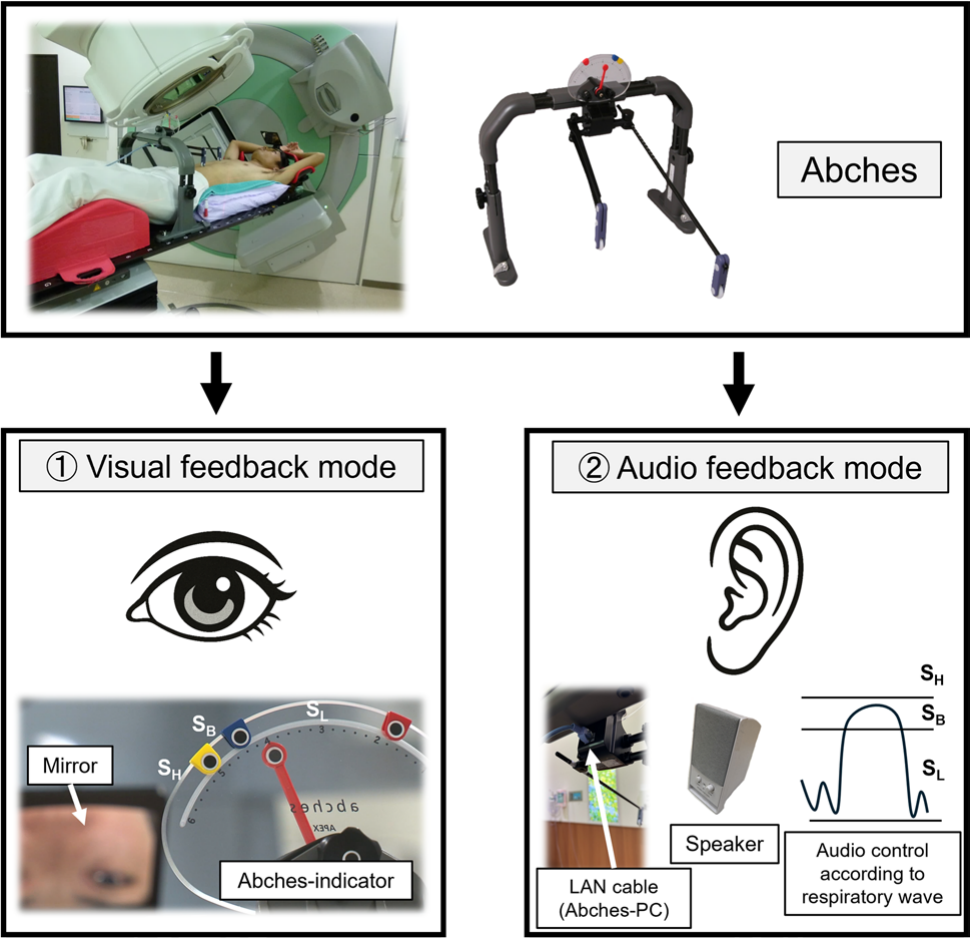
Appearance of the Abches system (top image). Respiratory signals—i.e., movements of the abdomen and chest—are detected via two mechanical fulcrums. The system supports two feedback modes: (1) visual feedback and (2) auditory feedback. In mode (1), the patient uses a mirror to visually monitor the indicator value and align their breathing accordingly (bottom left image). In mode (2), the Abches device is connected to a PC and speakers via a LAN cable, and the respiratory waveform is processed to provide auditory guidance through three distinct sound levels (bottom right image). The waveform is segmented into three amplitude-based regions, each associated with a specific cue: S_L_ (sound emitted when below the breath-holding range), S_B_ (within the breath-holding range), and S_H_ (above the breath-holding range)

The Abches system is a self-respiratory control device that utilizes visual cues for guidance. It consists of a main unit, an indicator panel, and two fulcrums placed on the patient’s chest or abdomen (shown in Fig. 2). A pointer connected to the indicator panel moves in tandem with the fulcrums during respiration. In visual feedback mode, a mirror positioned near the patient’s head allows them to observe their respiratory motion (bottom left image of Fig. 2). On the other hand, in audio feedback mode, the Abches device is connected to a PC and speakers via a LAN cable, and the respiratory waveform is processed to provide auditory guidance through three distinct sound levels (bottom right image of Fig. 2). The waveform is segmented into three amplitude-based regions, each associated with a specific cue: S_L_ (sound emitted when below the breath-holding range), S_B_ (within the breath-holding range), and S_H_ (above the breath-holding range). These sounds had different pitches (SL: slow pitch, SB: fast pitch, SH: a constant high-pitched sound), allowing patients to recognize them intuitively. Further information on the device is provided in previous reports, including its applications in DIBH for left-sided breast cancer and respiratory gating techniques with conventional linac system for chest and abdominal organs [14, 16, 17]. When adapting to these technologies, after performing patient setup as described above, connect the Abches device to the LAN cable and manage the respiratory waveform on the PC.

### Imaging and evaluation

CT scanning for Group A was performed using the HiSpeed DXi (GE Medical Systems, Milwaukee, WI, USA) with a 2 mm slice thickness and the following parameters: 120 kV, 200 mA, 1 s per helical rotation, a pitch of 1.5, and a 38 cm field of view covering the entire thorax or abdomen (512 × 512 pixels). Group B was scanned under the same conditions using the Aquilion LB (Canon Medical Systems, Japan). The average scan time was approximately 10–15 s per scan, with each patient required to hold their breath only once during CT acquisition.

**Fig. 3:**
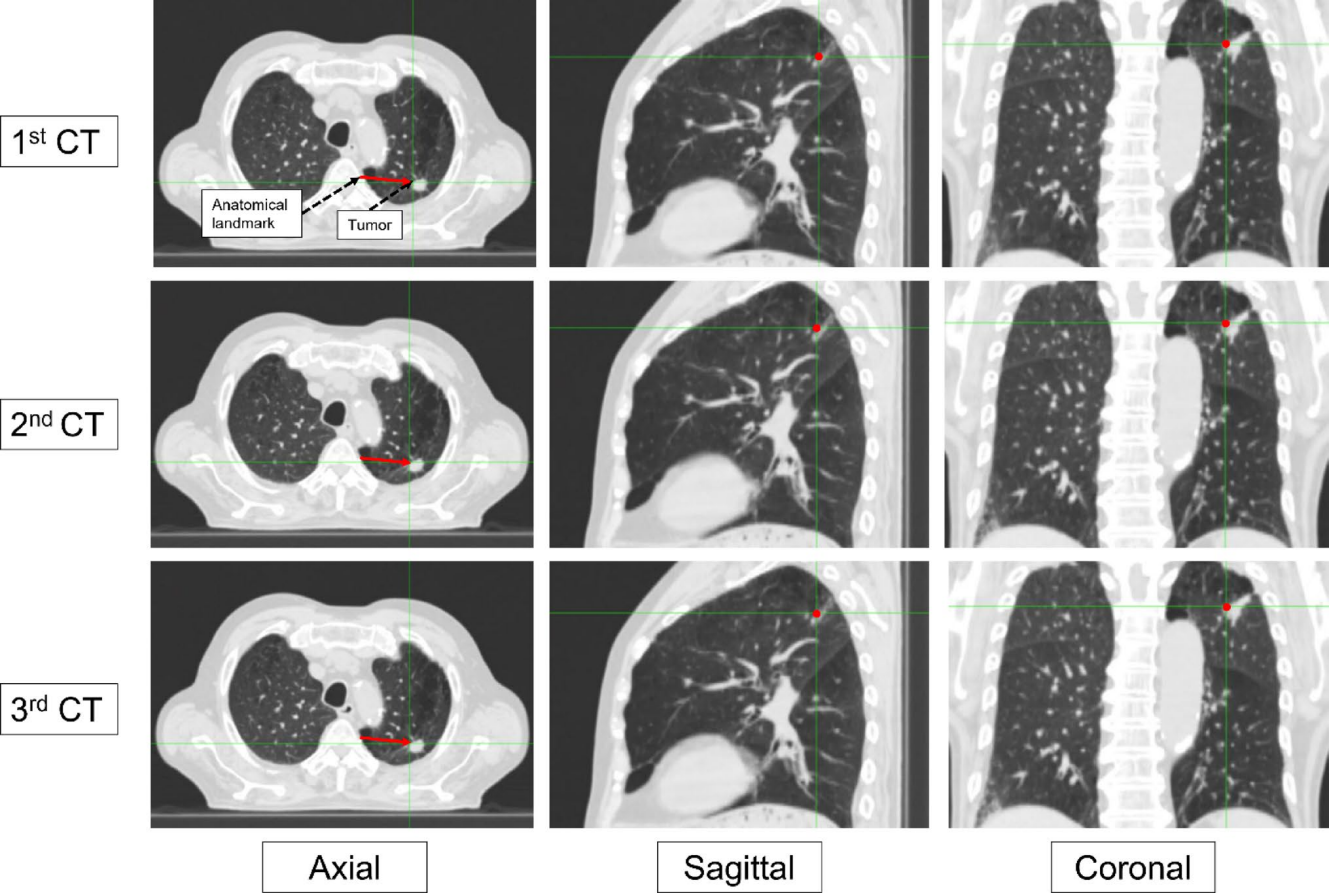
Three repeated CT images of a typical case are shown. From left to right, axial, sagittal, and coronal views are presented. In the axial view, the red arrow indicates the distance from an anatomical landmark to the tumor, while in the sagittal and coronal views, the red point denotes the same anatomical landmark relative to the tumor

The reproducibility of breath-holding under audio and visual guidance was assessed by measuring the distance from an anatomical landmark to the tumor across three repeated CT images for each method. Figure 3 shows three repeated CT scans of a representative case. The magnitude of displacement in each dimension (lateral, vertical, and longitudinal) as well as the three-dimensional vector were evaluated. After CT acquisition, patients were asked to indicate their preferred guidance method (i.e., visual or audio) for comfortable breath-holding. This survey employed a three-choice format, requiring participants to select one of the following options: auditory preference, visual preference, or either is acceptable. The correlation between the two guidance methods was also analyzed.

For statistical analysis, the normality of each dataset was assessed using the Shapiro–Wilk test. If the data followed a normal distribution, the Student’s t-test was applied; otherwise, the Wilcoxon test was used. Consequently, comparisons between the Audio and Visual groups were conducted using the Wilcoxon test, whereas comparisons between Group A and Group B were analyzed using the Student’s t-test. In addition, comparisons among the three groups based on patient preference (Audio, Visual, or Either) were performed using the Kruskal–Wallis test. A significance level of α = 0.05 was adopted for all statistical tests. All statistical analyses were conducted using JMP Pro version 18 (SAS Institute, Cary, NC, USA).

## Results

**Fig. 4:**
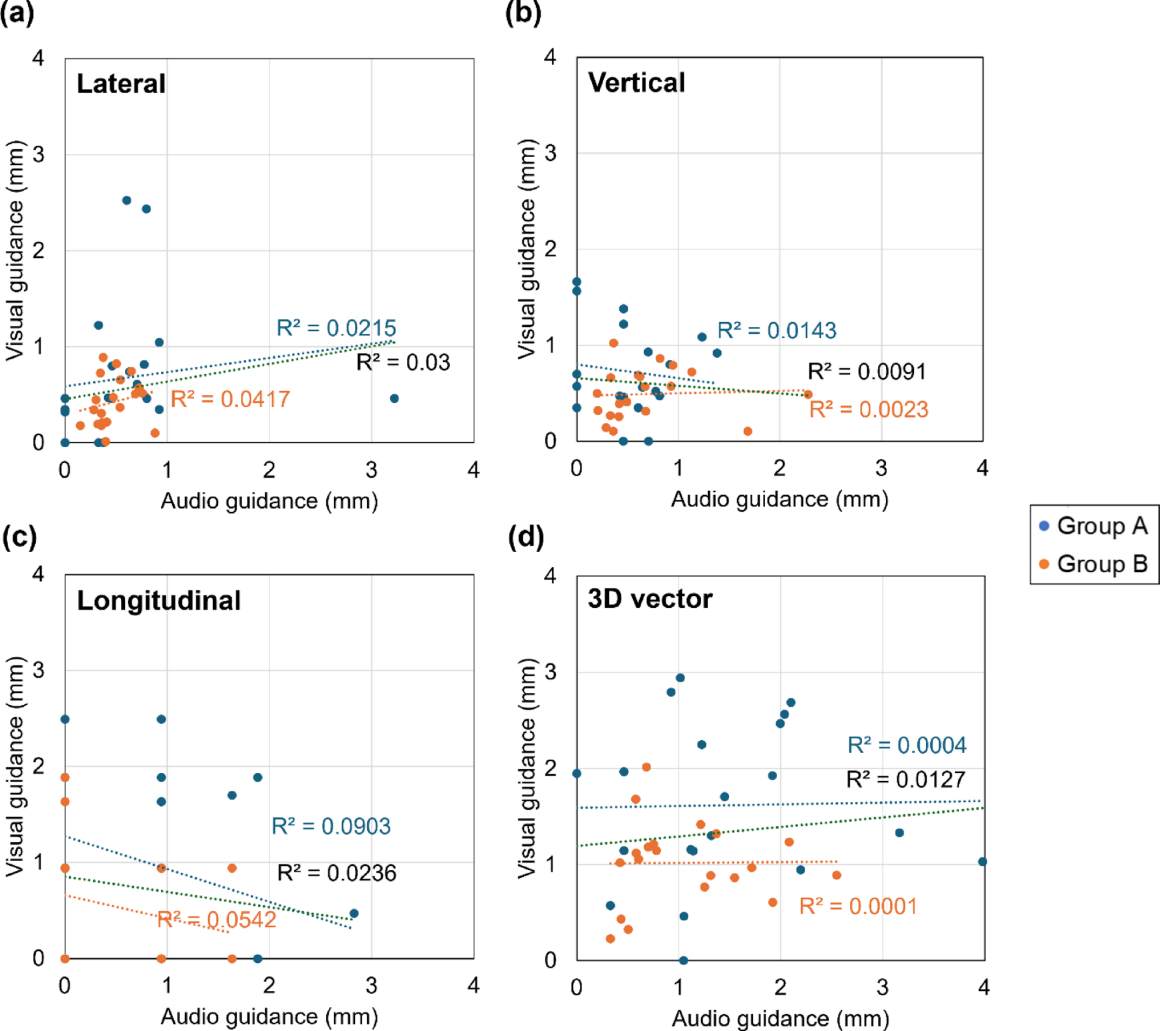
Breath-hold reproducibility in each direction (lateral, vertical, longitudinal) and the 3D vector for all 40 patients. Group A and Group B are represented in blue and orange, respectively. Correlation coefficients are indicated by colored text: blue for Group A, orange for Group B, and black for all patients

Figure 4 presents the breath-hold reproducibility in each direction and the 3D vector for all 40 patients, with Group A shown in blue and Group B in orange. Correlation coefficients were also calculated (Group A: blue text, Group B: orange text, all patients: black text), revealing no correlation between audio and visual guidance (R^2^ < 0.1). Table 2 summarizes the mean and range of breath-hold reproducibility for both guidance methods and the results of intergroup comparisons using the Wilcoxon test. For visual guidance, the median [IQR] deviations were 0.54 [0.35–0.79] mm vertically, 0.46 [0.21–0.73] mm laterally, and 0.94 [0.00–0.94] mm longitudinally, with a 3D vector deviation of 1.13 [0.60–1.77] mm. For audio guidance, the corresponding median [IQR] values were 0.48 [0.35–0.79] mm, 0.44 [0.33–0.70] mm, 0.94 [0.00–0.94] mm, and 1.13 [0.60–1.77] mm, respectively. Overall, both methods demonstrated high reproducibility, with average deviations less than 1 mm in each direction and less than 1.5 mm in the 3D vector. The range of variations was comparable between the two methods, and no statistically significant differences were observed in any direction or in the 3D vector (p > 0.05). In addition, although the Kruskal–Wallis test showed no significant differences among the patient-preference subgroups, a modest trend was observed in which the visual-preference group tended to show slightly smaller median deviations than the audio-preference group across most axes.

**Table 2:** Comparison of breath-hold reproducibility between visual and audio feedback using Kruskal–Wallis test and Wilcoxon-test

		Visual feedback				P-value†	Audio feedback					P-value* (Visual vs. Audio feedback)
		Patient preference					Patient preference					
		All (n = 40)	Visual (n = 22)	Audio (n = 12)	Either (n = 6)		All (n = 40)	Visual (n = 22)	Audio (n = 12)	Either (n = 6)	P-value†	
Vertical [mm]	Mean ± SD	0.61 ± 0.39	0.67 ± 0.39	0.58 ± 0.42	0.44 ± 0.19	0.2791	0.61 ± 0.46	0.58 ± 0.52	0.59 ± 0.31	0.75 ± 0.47	0.5064	0.9317
	Median [IQR]	0.54[0.35–0.79]	0.54[0.41–0.84]	0.57[0.22–0.85]	0.45[0.36–0.55]	–	0.48[0.35–0.79]	0.46[0.30–0.76]	0.54[0.40–0.73]	0.60[0.52–0.85]	–	–
Lateral [mm]	Mean ± SD	0.55 ± 0.53	0.60 ± 0.65	0.52 ± 0.37	0.41 ± 0.20	0.6037	0.53 ± 0.50	0.56 ± 0.64	0.50 ± 0.23	0.50 ± 0.24	0.8710	0.5654
	Median [IQR]	0.46[0.21–0.73]	0.46[0.24–0.69]	0.56[0.26–0.80]	0.40[0.23–0.54]	–	0.44[0.33–0.70]	0.44[0.31–0.65]	0.50[0.35–0.70]	0.35[0.33–0.63]	–	–
Longitudinal[mm]	Mean ± SD	0.73 ± 0.78	0.68 ± 0.85	0.67 ± 0.78	1.06 ± 0.26	0.2382	0.75 ± 0.75	0.74 ± 0.68	1.00 ± 0.88	0.31 ± 0.44	0.1765	0.9465
	Median [IQR]	0.94[0.00–0.94]	0.0[0.00–0.94]	0.24[0.00–1.18]	0.94[0.94–0.94]	–	0.94[0.00–0.94]	0.94[0.00–0.94]	0.94[0.00–1.70]	0.00[0.00–0.71]	–	–
3D vector [mm]	Mean ± SD	1.32 ± 0.72	1.40 ± 0.78	1.21 ± 0.72	1.25 ± 0.23	0.8980	1.26 ± 0.82	1.24 ± 0.89	1.37 ± 0.80	1.10 ± 0.43	0.6962	0.5257
	Median [IQR]	1.13[0.60–1.77]	1.14[0.89–1.94]	1.19[0.72–1.57]	1.20[1.14–1.29]	–	1.13[0.60–1.77]	1.08[0.49–1.82]	1.22[0.76–2.02]	1.06[0.71–1.43]	–	–

^†^, p-values by using Kruskal–Wallis test between 3 groups of patient preference, *: p-values by using Wilcoxon test between 2 groups of all patients for visual and audio feedback

Table 3 presents the comparison between Group A and Group B, conducted using the Student’s t-test with 95% confidence intervals. For visual guidance, Group B tended to show smaller deviations in all directions compared with Group A, with a significant difference observed in the 3D vector (mean ± SD: 1.62 ± 0.82 mm vs. 1.02 ± 0.42 mm; 95% CI [0.17, 0.86]; p = 0.007).Similarly, under audio guidance, Group B also exhibited smaller deviations, with a significant difference confirmed in the longitudinal direction (mean ± SD: 1.06 ± 0.78 mm vs. 0.45 ± 0.58 mm; 95% CI [0.16, 1.07]; p = 0.009).

**Table 3:** Comparison of breath-hold reproducibility and patient preference between Group A and Group B using Student’s t-test (*: significance)

	Group A (Visual ⇒ Audio) (n = 20)	Group B (Audio ⇒ Visual) (n = 20)	95% confidence interval	P-value (Group A vs. Group B)
*Visual guidance*				
Vertical [mm]	0.72 ± 0.46	0.49 ± 0.25	[− 0.01, 0.47]	0.0610
Lateral [mm]	0.68 ± 0.69	0.42 ± 0.25	[− 0.09, 0.59]	0.1385
Longitudinal[mm]	0.91 ± 0.89	0.55 ± 0.60	[− 0.14, 0.86]	0.1544
3D vector [mm]	1.62 ± 0.82	1.02 ± 0.42	[0.17, 0.86]	0.0072*
*Audio guidance*				
Vertical [mm]	0.53 ± 0.39	0.69 ± 0.51	[− 0.46, 0.13]	0.2726
Lateral [mm]	0.59 ± 0.68	0.47 ± 0.18	[− 0.21, 0.44]	0.4757
Longitudinal[mm]	1.06 ± 0.78	0.45 ± 0.58	[0.16, 1.07]	0.0092*
3D vector [mm]	1.45 ± 0.94	1.07 ± 0.62	[− 0.14, 0.90]	0.1489
Which is favor?	Visual: 13	Visual: 9		
	Audio: 6	Audio: 6		
	Both: 1	Both: 5		–

Although other directions did not reach statistical significance, most showed confidence intervals favoring Group B, suggesting a consistent trend toward improved reproducibility. These intergroup differences, however, may have been influenced by differences in treatment year (2009 vs. 2022) and equipment, rather than by methodological factors alone. Regarding patient preference between the two breath-hold methods, visual guidance was favored by more patients in both Group A and Group B. In total, 22 patients favored visual, 12 preferred audio, and 6 reported no preference.

## Discussion

Respiratory motion arises from an integrated neurophysiological loop involving brainstem rhythm-generating circuits [18] and cortical networks that mediate volitional modulation of breathing [19]. Sensory feedback from pulmonary and thoracic mechanoreceptors is rapidly processed at the cortical level, as evidenced by respiratory-related evoked potentials detected with high-density EEG [20]. Furthermore, respiration interacts dynamically with large-scale cortical oscillatory networks in a phase-dependent manner [21], and interoceptive processing shapes individual variability in respiratory control [22]. Within this neurophysiological framework, visual biofeedback has been shown to enhance breath-hold consistency during radiotherapy [23]. Visual cues typically provide continuous, actively monitored information that facilitates moment-to-moment self-adjustment, whereas auditory guidance tends to be intermittent and passively received, potentially engaging a distinct breath-hold control mechanism [24]. Given the potential variability in individual patients’ aptitude for different feedback modalities, a respiratory indicator system that incorporates both approaches in a complementary manner is likely to offer greater clinical utility. In this context, the Abches system is a uniquely capable device, as it already integrates both functions within a single platform. In our previous study, we reported that there was no correlation between auditory and visual guidance in terms of breath-hold reproducibility on a per-patient basis, suggesting that the optimal guidance method should be determined individually [15]. However, a major limitation of that study was the fixed order in which the guidance methods were applied. In the present study, we addressed this limitation by including a new cohort in which the order of guidance was reversed, thereby enabling a more balanced comparison. The results were largely consistent with those of the previous study, further reinforcing the reliability of our earlier findings.

The utility of audio-visual biofeedback in respiratory-gated radiotherapy has been explored in several previous studies [23, 25]. George et al. investigated the effects of combined audio-visual feedback on respiratory-gated treatment and demonstrated that it effectively reduced intra-gating respiratory motion, as measured by the standard deviation of respiratory signals within the gating window [23]. Venkat et al. compared two types of visual feedback displays—a bar model and a wave model—and found that the wave display achieved over 50% and 70% reductions in displacement and period variability, respectively, compared with free breathing [25]. Similarly, Nakajima et al. assessed visual and audio-visual feedback using the Abches system in healthy volunteers and reported that both feedback modes improved respiratory stability compared with no feedback; however, direct comparisons between audio and visual guidance were not performed [26]. While these studies demonstrated the value of biofeedback for respiratory control, most focused on free-breathing or respiratory-gated treatments rather than voluntary breath-hold. Differences in study design, feedback methods, and monitoring systems also make direct comparison difficult. The present study extends previous work by directly comparing auditory and visual guidance during breath-hold in a clinical setting.

Our current study revealed no significant differences of breath-hold reproducibility between preferences of guidance methods. In this study, 55% of patients (22/40) preferred visual guidance, 30% (12/40) preferred auditory guidance, and 15% (6/40) reported no preference. As there was no significant difference in breath-hold reproducibility between the visual and auditory methods, visual coaching appears to be the most appropriate option for approximately 70% of patients. However, considering that patients with visual impairments and roughly 30% of all participants preferred auditory feedback, allowing patient-specific selection may facilitate better continuity and adherence to breath-hold radiotherapy.

Although the geometric differences observed in this study are small and, based on prior SBRT simulation and phantom studies, are expected to translate into only modest changes—typically ≤ 1% per millimeter in volume-based target dose metrics—across most clinical scenarios [27–29]. However, regions with very steep dose gradients, such as PTV–OAR interfaces, can still exhibit voxel-level dose differences on the order of 1–2 Gy per millimeter of displacement. Accordingly, even submillimeter to millimeter-scale uncertainties should be managed carefully when high-gradient regions are adjacent to critical organs.

Recent systematic reviews have also highlighted the considerable inter-patient variation in breath-hold stability and reproducibility, particularly in upper abdominal radiotherapy. Farrugia et al. summarized data from 41 studies and reported a median cranio-caudal inter-fraction reproducibility of 0.6 mm in exhale breath-hold and greater variability in DIBH [30]. Our findings were largely consistent with these results. However, they noted that no single technique emerged as clearly superior, as the reported confidence intervals overlapped and the study methodologies differed in breath-hold definitions, imaging modalities, surrogate markers, and analysis criteria [30].

Although audio guidance was more frequently preferred by patients, preference did not consistently correlate with improved breath-hold reproducibility. Guidance selection based on post-CT image analysis yielded slightly better reproducibility than patient preference (mean difference < 0.5 mm per axis). While the magnitude of this difference is small and unlikely to have a major clinical impact in most conventional settings, it highlights the potential importance of objective evaluation using CT images over subjective preference when selecting guidance modes. As emphasized in AAPM TG-76, patient engagement and training are essential for effective respiratory management [31]. Accordingly, we advocate for a patient-centered approach, wherein the guidance modality is tailored based on both imaging feedback and individual patient characteristics.

There are several limitations to the present study. First, there were notable differences between the 2009 and 2022 cohorts, with the latter demonstrating improved accuracy (Table 3). Several clearly identifiable technological advancements likely contributed to this improvement. One factor was the enhancement of CT image quality: the Hi Speed DX/i used in 2009 employed a single–detector-row system, whereas the Canon Aquilion LB used in 2022 had a 16-detector-row configuration, enabling more precise respiratory positional measurements. In addition, improvements in the operability of the Abches control software, along with nearly a decade of accumulated staff experience with Abches-based breath-hold techniques, may have further contributed to the superior performance observed in the 2022 cohort.

Second, the study included a mix of inhalation and exhalation breath-hold techniques, with inhalation cases being predominant. For example, Kimura et al. reported that the end-expiration phase was more stable than the end-inspiration phase with respect to breath-hold reproducibility [32]. Therefore, further investigation focusing on exhalation breath-holds with a larger sample size is warranted. Third, regarding the auditory–visual system evaluated in this study, the optimal approach must be tailored to each patient, and therefore it does not inherently improve efficiency. Nonetheless, our findings show that both techniques can achieve acceptable breath-hold accuracy. Accordingly, for patients who cannot use one modality (e.g., those who are totally blind or have hearing impairment), the other can serve as a practical alternative. Devices that incorporate both modalities remain limited, and we believe that this dual functionality offers meaningful clinical benefit.

## Conclusion

Regarding the reproducibility of breath-holding, both auditory and visual guidance methods demonstrated high precision. However, no significant differences were observed between the two approaches, and patient preference did not appear to affect reproducibility.
